# Society Meetings

**Published:** 1898-12

**Authors:** 


### SOCIETY MEETINGS.

THE third Pan-American Medical Congress has been postponed, as will appear by the following correspondence : International Executive Commission of the Pan-American Medical Congress, office of the secretary, Cincinnati, November 5, 1898. My dear Sir—I have the honor to announce that in April, 1898, I received from Dr. José Manuel de los Rios, chairman of the committee on organisation of the third Pan-American Medical Congress, a request that, in consequence of the then existing rebellion in Venezuela, no definite arrangements be made at that time relative to the meeting of the congress previously appointed to be held in Caracas in December, 1899. The following communication relative to the same subject is just at hand : Caracas, September 25, 1898. Dr. Charles A. L. Reed, Secretary of the International Executive Commission, Cincinnati, Ohio. Dear Sir—After having sent my communication, dated April last, I find it to be my duty to notify you that, although the considerations pointed out in it have already ended, our country has been scourged by small-pox, which has taken up all our physicians' activities and time, depriving them of going into scientific work. As that state of mind of our people and government, after such calamities as war and epidemic, would greatly interfere with the good success of our next meeting, I beg leave to tell you, in order that you will convey it to the International Executive Committee, that our government and this commission would be grateful to have the meeting which was to take place in Caracas in December, 1899, adjourned to one year later. I am, dear doctor, yours respectfully, The President. (Signed) Dr. José Manuel de los Rios. In accordance with the request of the government of Venezuela, and of the committee on organisation, the third Pan-American Medical Congress is hereby postponed to meet in Caracas in December,

1900. For the International Executive Commission, Charles A. L. Reed, Secretary.

---

THE Buffalo Academy of Medicine held meetings during the month of November, 1898, as follows:

*Section on Surgery.*—Tuesday evening, November 1st, program: Gonorrhea, by W. H. Heath; discussed by Drs. Harrington and Green. Surgical kidney, by M. Hartwig; discussed by Drs. Park, Heath, Parmenter and others.

*Section on Medicine.*—Tuesday evening, November 8th, program: The hypertrophic form of infantile palsy, by Dr. L. Pierce Clark, Craig Colony, Sonyea, N. Y. Myxedema, acromegaly and giantism, by Floyd S. Crego. Some etiological factors of nervous diseases, by James W. Putnam.

*Section on Pathology.*—Tuesday evening, November 15th, program: Carcinoma of the duodenum, by Charles D. Aaron, of Detroit, Mich. On the origin of vertigo, by Charles G. Stockton.

*Section on Obstetrics.*—Tuesday evening, November 22d, program: Multiple pregnancy, by Thomas F. Dwyer. Use of Catgut in Surgery, by C. C. Frederick. Placenta previa, specimen presented, by Dr. Brennen, interne of Erie County Hospital.

---

THE Lake Erie Medical Society held its regular quarterly meeting at Dayton, N. Y., November 11, 1898. The following program was observed: Albuminuria in pregnancy, W. J. French, M. D., Hamlet, N. Y.; Presentation of cases by several members; Coccygodnia, J. E. Holden, M. D., Cattaraugus, N. Y.; Subject unknown, Fred Beals, M. D., Salamanca, N. Y.

---

THE New York State Association of Railway Surgeons elected the following officers at its last meeting: President, Theodore D. Mills, Middletown; vice-president, J. L. Eddy, Olean, and G. N. Hall, Binghamton; secretary, C. B. Herrick, Troy; treasurer, H. P. Jack, Canisteo.

---

THE American Public Health Association at its last meeting elected the following officers for the ensuing year: President, George H. Rohé, Sykesville, Md.; first vice-president, Henry Mitchell, Asbury

Park, N. J.; second vice-president, J. E. Monjaras, San Luis Potosi, Mexico; secretary, C. O. Probst, Columbus, O.; treasurer, Henry D. Holton, Brattleboro, Vt.

---

THE Esculapian Club, of Buffalo, will hold its December meeting at the house of Dr. Charles E. Congdon, 1034 Jefferson street, at which Dr. W. B. Saltsman will read a paper.

